# Quasi suppression of higher-order diffractions with inclined rectangular apertures gratings

**DOI:** 10.1038/srep16502

**Published:** 2015-11-13

**Authors:** Yuwei Liu, Xiaoli Zhu, Yulin Gao, Wenhai Zhang, Quanping Fan, Lai Wei, Zuhua Yang, Qiangqiang Zhang, Feng Qian, Yong Chen, Weihua He, Yinzhong Wu, Zhuoyang Yan, Yilei Hua, Yidong Zhao, Mingqi Cui, Rong Qiu, Weimin Zhou, Yuqiu Gu, Baohan Zhang, Changqing Xie, Leifeng Cao

**Affiliations:** 1 Research Center of Laser Fusion, National Key Laboratory of Laser Fusion, CAEP, P.O. Box 919-986, Mianyang, 621900, P. R. China; 2 Joint Laboratory for Extreme Conditions Matter Properties, Southwest University of Science and Technology and Research Center of Laser Fusion, Mianyang 621900, P. R. China; 3 Key Laboratory of Microelectronics Devices and Integrated Technology, Institute of Microelectronics, Chinese Academy of Sciences, Beijing, 100029, P. R. China; 4 Institude of High Energy Physics, Chinese Academy of Science, Beijing, 100039, P. R. China

## Abstract

Advances in the fundamentals and applications of diffraction gratings have received much attention. However, conventional diffraction gratings often suffer from higher-order diffraction contamination. Here, we introduce a simple and compact single optical element, named inclined rectangular aperture gratings (IRAG), for quasi suppression of higher-order diffractions. We show, both in the visible light and soft x-ray regions, that IRAG can significantly suppress higher-order diffractions with moderate diffraction efficiency. Especially, as no support strut is needed to maintain the free-standing patterns, the IRAG is highly advantageous to the extreme-ultraviolet and soft x-ray regions. The diffraction efficiency of the IRAG and the influences of fabrication constraints are also discussed. The unique quasi-single order diffraction properties of IRAG may open the door to a wide range of photonic applications.

Diffraction gratings with periodic structures are simple and fundamental optical elements that disperse incident light into its constituent spectrum^1^,^2^. They also play a crucial role in the extreme ultraviolet and x-ray regions. Their applications range from laboratory-generated and astrophysical plasma diagnosis to synchrotron radiation light monochromator^3^,^4^,^5^,^6^,^7^. From its very beginning, conventional binary (black and white) transmission gratings (TGs) are composed of lots of evenly spaced parallel slits on an opaque screen. Most applications of diffraction gratings have only relied on the generated ±1st diffracted orders. The broadband spectra measured by TGs always suffer from higher-order diffraction contamination due to the higher-order overlapping^8^. Consequently, complicated unfolding process is a cumbersome task and always inevitable to filter the stray light, resulting in ambiguous spectroscopic data^9^. Similarly, the synchrotron radiation monochromatic light obtained with conventional diffraction gratings also contains unwanted higher-order harmonic components, a complicated higher-harmonics suppressor system is needed to reduce higher-order diffraction^9^,^10^,^11^.

Sinusoidal amplitude transmission grating (STG) with continuous relief can suppress the higher-order diffraction effectively in the visible light region^8^. The robust fabrication process of imprinting and dry etching can ensure production quality of STG in the visible light region^12^. However, the diffraction properties of the diffractive optical elements in the extreme ultraviolet and x-ray regions differ from those in the visible light region. This is due to the fact that all known materials in the extreme ultraviolet and x-ray regions have complex refractive index and is very close to unity. It is difficult to fabricate high-Z metal absorber with the required continuous relief using modern photolithography technology. Single optical element in the extreme ultraviolet and x-ray regions typically consists of a thin membrane that is transparent to X-rays, on which x-ray absorber (usually gold) patterns are placed^13^. For certain applications such as x-ray astronomy and diagnostics of laser produced plasmas, free-standing gold bars are often required^14^. Thus, the manufacture of a STG for the extreme ultraviolet and x-ray regions remains a great challenge^15^. In order to overcome the so-called higher-order diffractions contamination problem, several techniques have been developed in the past years, e.g., the use of thin films to reduce the influence of higher-order effects^16^, the amplitude grating whose stripes present rough edges^17^ and the order-sorting method using an array detector^18^. In 2007, Cao *et al.* proposed the concept of binary sinusoidal transmission grating (BSTG)^11^. A BSTG consists of sinusoidal-shaped apertures in two dimensions. An amplitude sinusoidal transmission function along one dimension can be realized by such structures. Both the calculated and experimental results demonstrated that the BSTG can eliminate higher-order diffraction effectively along the symmetric axis. Followed by this new idea, a variety of single-order diffraction gratings were then further developed, such as quantum-dot-array diffraction gratings (QDADG)^19^, zigzag transmission gratings (ZZTG)^20^, quasi-sinusoidal single-order diffraction transmission gratings (QSTG)^21^ and modulated groove position gratings (MGPGs)^22^. All these efforts attempted to realize a sinusoidal amplitude transmission function in one dimension by arranging special geometric patterns in two dimensions, resulting in a sinusoidal average diffractive effect that suppresses higher-order diffractions. However, for the purpose of realizing free-standing metal structures, support struts are required to satisfy both optical and mechanical requirements, resulting in a loss of 25–50% of the grating throughput. Such support struts not only produce diffraction spikes in the diffraction plane^23^, but also decrease the diffraction efficiency (the ratio between the intensity of the 1st or −1st order diffracted light versus the intensity of the incident light). Until now, the above mentioned gratings with high line density (≥1000 lines/mm) and free-standing structures have not been reported.

On the basis of our previous work, here we introduce a new type of single optical element with the capabilities of quasi-single order diffraction and free-standing structures. The key idea is to rotate regular rectangular apertures to 45 degree, leading to dominant ±1st diffraction orders on the observation line. Compared with the previous schemes, this simple and compact single optical element, named inclined rectangular aperture gratings (IRAG), consists of a series of periodically arranged inclined rectangular apertures milled into Au film with thickness ranges from few tens of nanometers to a few microns. Such diffractive structures allow one to suppress the high-order diffraction effect. Furthermore, free-standing gold structures for the extreme ultraviolet and x-ray regions can be achieved without the need of any support strut. An IRAG on a quartz substrate for the visible light region was fabricated using electron beam direct writing technique. The measurement results show good agreement with the theoretical predictions. By combing electron beam lithography and x-ray lithography, a free-standing x-ray IRAG with line density of 1000 lines/mm was also fabricated. The optical characterization that was performed at Beijing synchrotron radiation facility demonstrates the effectiveness of this method.

## Results

### Inclined rectangular aperture gratings concept

**Figure 1:**
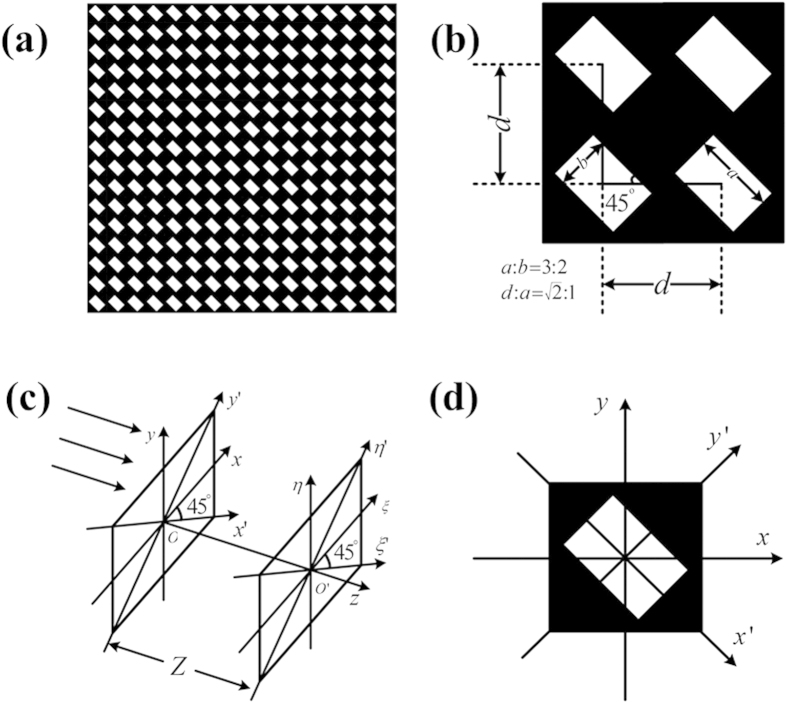
Inclined rectangular aperture gratings scheme. (**a**) Schematic diagram of an IRAG. (**b**) The details of four inclined rectangular apertures. (**c**) The coordinate systems in the aperture plane and observation planes. (**d**) The structure of an inclined rectangular aperture.

Figure 1 shows the schematic illustration of an IRAG. Here, by rotating the coordinate system X-Y clock wise with a 45 degree angle, a coordinate system X’-Y’ is generated. The periodically arranged inclined rectangular apertures which generate diffracted orders are perforated on an Au film. Since the IRAG has no connected regions, the free-standing metal structures fabrication would be highly advantageous. The size of the aperture is a×b and *a* : *b* = 3 : 2. The period *d* of the IRAG is equal to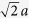. The coordinate transforming relations between the two coordinate systems can be expressed as


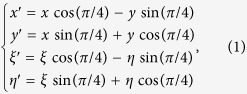


where (x, y) and (x′, y′) denote the grating plane, and (ξ, η) and (ξ′, η′) denote the observation plane, respectively.

### Theoretical analysis

Let us begin with an amplitude type IRAG which is commonly used in the visible light region. In the (x, y) coordinate system, the transmission function 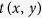 can be expressed as


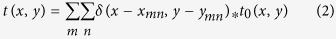


where (*x*_*mm*_, *y*_*mn*_) stand for the central position of the inclined rectangular apertures, and *t*_0_(*x*, *y*) is the transmission function of the elemental aperture. In the (x′, y′) coordinate system, the transmission function can be expressed as


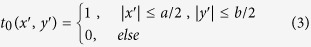


For simplicity and clarity, we only consider the case that the IRAG is normally illuminated by a monochromatic plane wave with amplitude of *A*_0_. The distribution of complex field in the observation plane can be obtained by the Fraunhofer diffraction formula


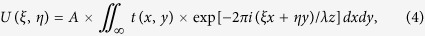


where 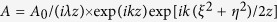, 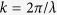 is the wave number, and *z* is the propagation distance between the single optical element plane and the observation plane.

By substituting Eq. (1)–Eq. (3) into Eq. (4), we obtain


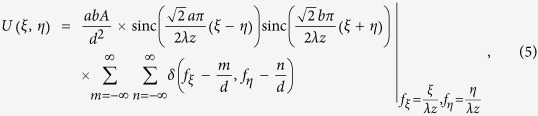


where 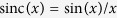.

For the purpose of suppressing higher-order diffraction, here we choose 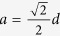 and 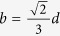. For this case, the intensity distribution of the diffraction pattern can be expressed as


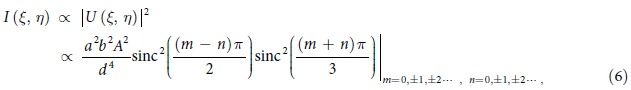


where *m and n* are the grating diffraction orders (spectral orders). The ±1th orders mean the diffraction order with *m* = 0, *n* = ±1 or *m* = ±1, *n* = 0. Similarly, the ±2th orders mean the diffraction order with *m* = 0, *n* = ±2 or *m* = ±2, *n* = 0, and so on. Here, we define a suppression ratio of higher-order diffractions of the IRAG as the ratio between the intensity of the higher-order diffracted light versus the intensity of the 1st or −1st order diffracted light. The binarization of the IRAG can significantly suppress the higher-order diffraction background. The suppression ratio of the ±5th and ±7th diffracted light is as low as −55.9 dB and −68 dB, respectively. Thus, as expected, an IRAG can obtain quasi-single-order diffraction effect in the observation plane, approaching that of a perfect STG.

Diffraction efficiency is also an important figure of merit of the IRAG. For a perfect IRAG, the absolute diffraction efficiency of the 0th order, ±1st orders, ±5th orders and ±7th are 11.11%, 3.08%, 0.0049% and 0.00123%, respectively, while the absolute diffraction efficiencies of other higher-order diffractions approach zero. The relative diffraction efficiency of the ±1st orders (the 1st or −1st order diffracted light intensity divided by the 0th order diffracted light intensity) is 27.72%.

Next we consider the case of the extreme-ultraviolet and x-ray regions. As mentioned above, even for a high-Z metal absorber, the interaction between the incident x-ray beam and the absorber is weak. Thus, Eq. (3) is rewritten as


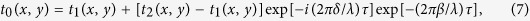


with


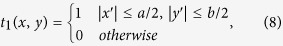



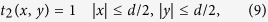


where 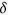 is the refractive index decrement, *β* represents the absorption index of the field amplitude, *λ* is the incident light wavelength, and τ is the absorber thickness.

By substituting Eqs (1) and (7) into Eq. (4), we obtain


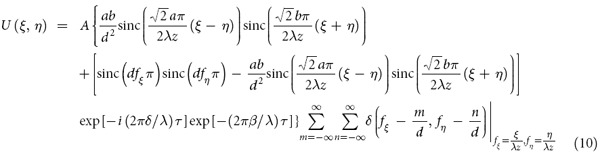


so


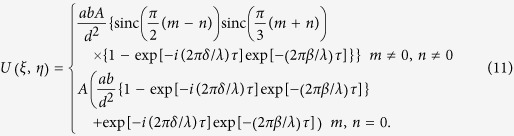


In the extreme-ultraviolet and soft x-ray regions, if the absorber thickness is thick enough, the diffraction properties of the IRAG are the same as those in visible light region. However, in the hard x-ray region, the radiation passes through the IRAG and is partially attenuated and also phase-shifted. Both the absorption and phase shift decrease with the wavelength, the diffraction efficiency will depend on the absorber thickness. Conventional transmission gratings with a duty cycle of 0.5 can achieve a maximum efficiency in each ±1 order of 10.13%. Here the first order diffraction efficiency of the IRAG is approximately 3%. Advances in high brilliance x-ray sources from laser-fusion targets and the high sensitivity x-ray sensors will allow efficient use of IRAG. In fact, the use of single optical element with low diffraction efficiency, such as photon sieve, is also applicable on acceptable timescales with present-day synchrotron light sources^24^. It should be noted that x-ray free electron (the fourth-generation synchrotron light) can produce x-ray beam with peak brightness 10^9^ times higher than that of the most powerful third-generation synchrotron light^25^. There will be a great opportunity to use the IRAG. However, for some special application with ultralow signal such as x-ray astronomy, IRAG with multilevel phase profiles should be designed and fabricated to improve the first order diffraction efficiency. In addition, the concept of critical angle transmission gratings which eliminates absorption concerns can also be applicable to our IRAG for achieving much higher diffraction efficiency^26^.

The above discussions are based on the assumption that the grating size is infinite. It is obvious that this is an ideal case. In practice, the gratings is finite in extent, and the distance between the grating plane and the observation plane is also finite, thus making the diffraction pattern different from that described by Eq. (6) or Eq. (11). Numerical simulation based on Kirchhoff-Fresnel diffraction formula was carried out to evaluate the diffraction property of the IRAG with finite size. The complex amplitude distribution in the observation plane can be obtained by Fresnel diffraction formula


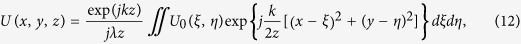


where 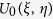 denotes the initial complex amplitude in the single optical element plane.

**Figure 2:**
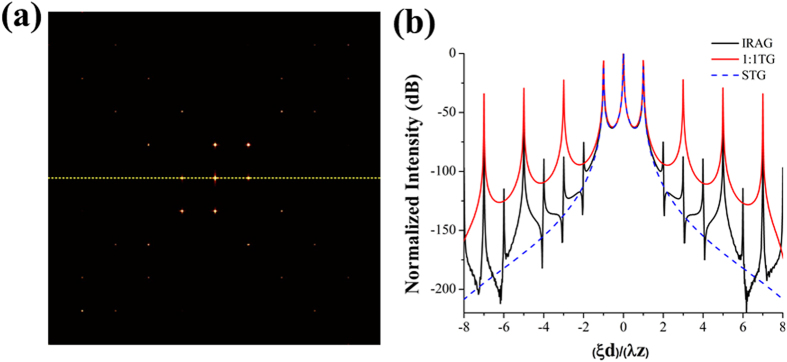
Numerical results on diffractive properties of an IRAG. (**a**)The far-field diffraction pattern of the IRAG. (**b**) Comparison of intensity profiles produced by an IRAG, a TG with duty cycle (ratio of grating line width to period) of 0.5, and a STG. The period of all gratings is the same and equal to 1 μm. The wavelength of the incident light is 5 nm.

The numerical results are shown in Fig. 2. The whole size of the IRAG is 50 μm × 50 μm, finite propagation distance between the grating plane and the observation plane is 1 m, the wavelength of the incident light is 5 nm and gold was chosen as the absorber material for its high stability and the large absorption coefficient in the soft x-ray region. Similar to the ideal case, the higher-order diffractions are significantly suppressed and close to the ideal sinusoidal transmission grating. The suppression ratios of the ±2nd, ±3rd, ±4th, ±5th, ±6th, and ±7th diffracted light are −67 dB, −79 dB, −81 dB, −50 dB, −106 dB and −64 dB, respectively.

### Experimental demonstration

**Figure 3:**
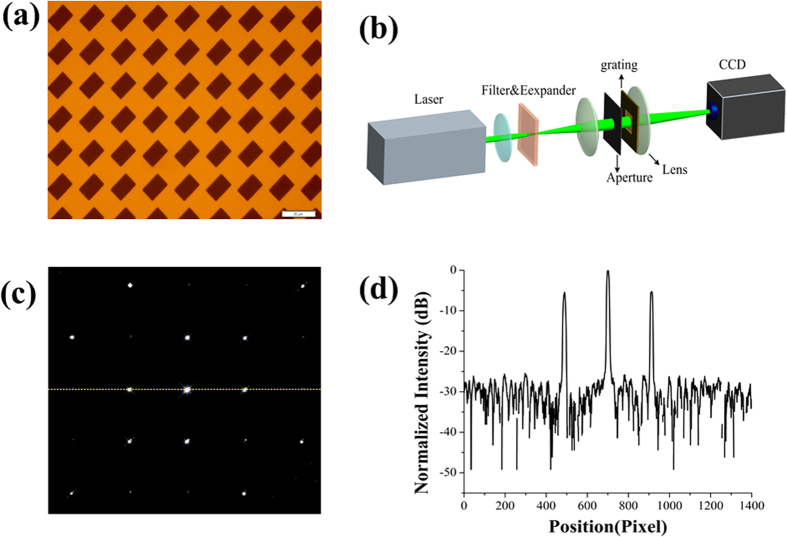
The fabricated IRAG in the visible light region. (**a**) Microscopy image of the fabricated IRAG. (**b**) Experimental system for optical demonstration. (**c**) Diffraction pattern recorded by the CCD camera. (**d**) The intensity distribution along the η axis.

In order to test the feasibility of the higher-order diffractions suppression in the visible light region, we firstly fabricated a binary IRAG with period of 20 μm by using an electron beam mask writer (MEBES4700S). A collimated laser beam with wavelength of 532 nm was used to illuminate the fabricated IRAG, and a charge coupled device (CCD) camera (Lumenera’s Lw230) with 1612 × 1214 pixels and 4.4μm pixel size was placed in the far field to record the diffraction patterns. The recorded results are shown in Fig. 3. As expected, the higher-order contributions of the fabricated IRAG are significantly suppressed. The measured suppression ratios of the ±2nd, ±3rd, ±4th, ±5th, ±6th and ±7th diffracted light are −54.9 dB, −71 dB, −76 dB, −72.4 dB, −79.2 dB, −68.9 dB, respectively. The corresponding theoretical calculated values are −67 dB, −79 dB, −81 dB, −50 dB, −10 6dB and −64 dB, respectively. This result verifies that the IRAG with quasi suppression of higher-order diffractions is superior to a conventional transmission grating.

**Figure 4:**
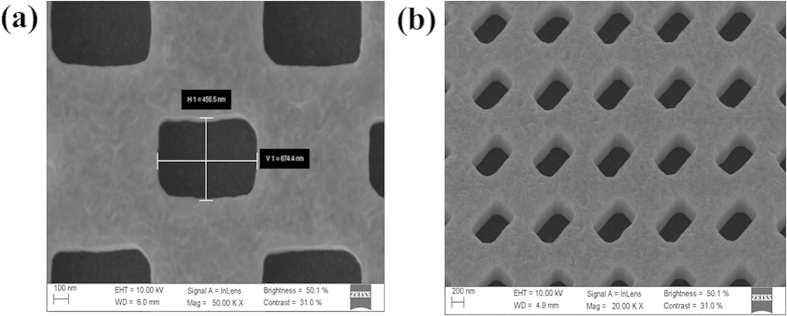
The fabricated free-standing x-ray IRAG with line density of 1000 lines/mm. (**a**) Scanning electron microscope image. (**b**) Amplified scanning electron microscope image of (**a**).

We have further investigated the feasibility of the IRAG in the soft x-ray region. As stated above, the material absorption in the soft x-ray region is much higher than that in the visible light region, and the free-standing structures are preferred. In this work, we fabricated a free-standing x-ray IRAG with line density of 1000 lines/mm, as shown in Fig. 4. The measured length *a* and width *b* are 674.4 and 455.5 nm, respectively. The corresponding CD bias are 32.7 and 15.9 nm, respectively. Details of the free-standing structures fabrication process can be found elsewhere^27^. In brief, there are two main steps in our approach. The first step is to pattern the IRAG structures with small thickness on an x-ray mask using 100 kV electron-beam lithography, followed by gold electroplating. The final step is to efficiently replicate IRAG using x-ray lithography. One major advantage of the IRAG is that no support strut is needed for the formation of free-standing structures. Thus, no additional lithography step is needed to fabricate a large-scale gold mesh as the support struts. The technical parameters of the fabricated free-standing x-ray IRAG are listed in Table 1.

**Figure 5:**
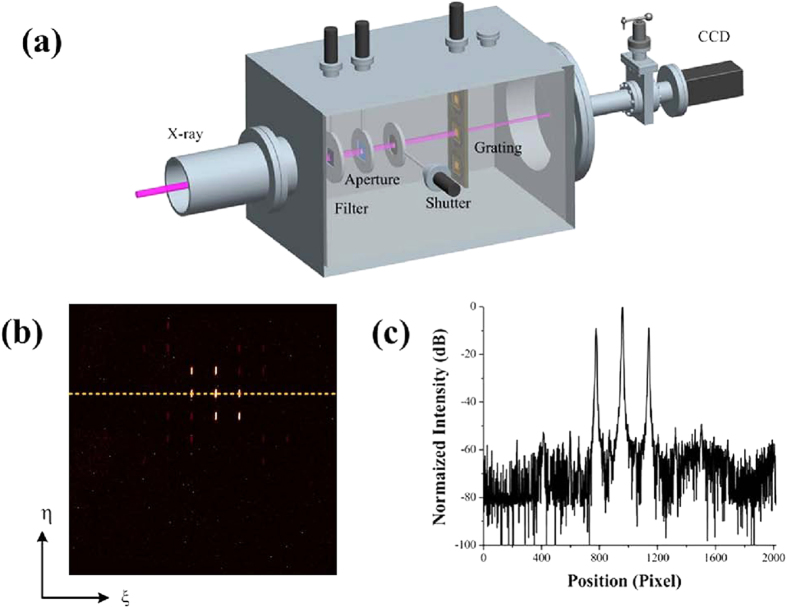
Experimental results of the fabricated free-standing x-ray IRAG. (**a**) The experimental arrangement. The CCD has an image area of 2048 × 2048pixels with a 13.6 μm square pixel size. The distance between the grating and the CCD is about 60 cm. The grating width and length are 100 μm and 600 μm, respectively. The Au absorber thickness is 550 nm. (**b**) The diffraction pattern of the fabricated IRAG at photon energy of 300 eV recorded by the CCD. (**c**) The integrated diffraction intensity of the diffraction orders along the ξ axis.

The performance of the fabricated free-standing x-ray IRAG was tested at 4B7B beamline of Beijing synchrotron radiation facility, which covers the photon energy range from 50 eV to 1700 eV and is designed for applications to soft x-ray spectroscopy and x-ray magnetic circular dichroism. The experimental setup is shown in Fig. 5(a). The beamline is equipped with a monochromator which consists of a varied line space grating and a flat mirror. A filter assembly which consists of an aluminum membrane with thickness of 0.2 μm and a carbon membrane with thickness of 1μm was mounted after the monochromator. The spot size on the measured free-standing x-ray IRAG was about 1 mm × 1 mm in area.

The measurement of absolute diffraction efficiency in the x-ray region is much more difficult than that in the visible and UV regime, especially at the first generation synchrotron radiation facility. This is because the synchrotron radiation experiments are set up parasitically with a primary interest in high-energy physics. Here we only recorded the diffraction pattern using a CCD camera with 2048 × 2048 pixels and 13.6 μm pixel size. The result is shown in Fig. 5(b). The normalized 1D profile of the diffraction pattern along η axis is shown in Fig. 5(c). The relative diffraction efficiencies of the −1st order and 1st order are 35.2% and 35.9%, respectively. The measured suppression ratios of the −2nd, −3rd, 2nd and 3rd are −43.1 dB, −43.6 dB, −41.6 dB and −40.3 dB, respectively. The measured suppression ratios of other higher-order diffraction can be suppressed below −46 dB. The experimental result differs slightly from the ideal intensity distribution shown in Fig. 2(b). This may be attributed to the limited dynamic range and signal-to-noise ratio of the CCD, filter inhomogeneity, slightly tilted installation of the measured IRAG and the inevitable imperfection of the fabrication. Nevertheless, the experimental results clearly demonstrate the effectiveness of our IRAG.

## Discussion

As previously shown, a slight deviation between analytical and experimental results is observed, which could be attributed to the limited dynamic range and signal-to-noise ratio of the CCD, filter inhomogeneity, slightly tilted installation of the measured IRAG and the inevitable imperfection of the fabrication. In the following, we will discuss the influences of practical fabrication errors on the IRAG dispersion performance. From a fabrication point of view, there are three main factors that can occur, including Au absorber thickness, position accuracy and critical dimension (CD) bias. Let us analyze and evaluate their influences on the IRAG dispersion performance one by one. First, for the present fabrication method, the Au absorber thickness of the fabricated IRAG is as large as 550 nm, the resulting transmission ratio of the incident x-ray photons with photon energy of 300 eV is as low as 8.3 × 10^−8^. Thus, here the fabricated IRAG can be considered to be of amplitude type, and the influence of Au absorber thickness on the practical performance can be neglected. Secondly, for the free-standing x-ray IRAG with line density of 1000 lines/mm presented in this work, the nominal position accuracy determined by the electron beam mask writer is about ±20 nm, which has little negative effect on the practical performance of IRAG. For the IRAG with higher line density, it should be noted that the position accuracy of 1.6 ± 0.6 nm can be achieved by state-of-the-art lithography tools^28^, which should satisfy the position accuracy requirement of IRAG. Finally, we analyzed and evaluated the influence of the CD bias of the inclined rectangular apertures on the practical performance. Here we define a relative CD bias as 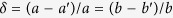, where 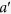 and 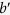 are the length and width of the fabricated inclined rectangular aperture, respectively, while *a* and *b* are the corresponding target CDs. Based on Eq. (6), the relative diffraction efficiency of the ±1st orders and the suppression ratios of the higher-order diffractions can be expressed as


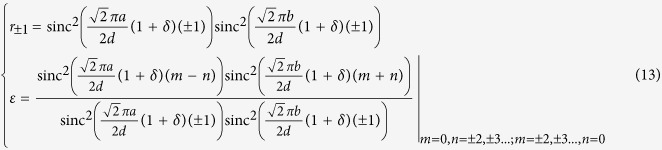


**Figure 6:**
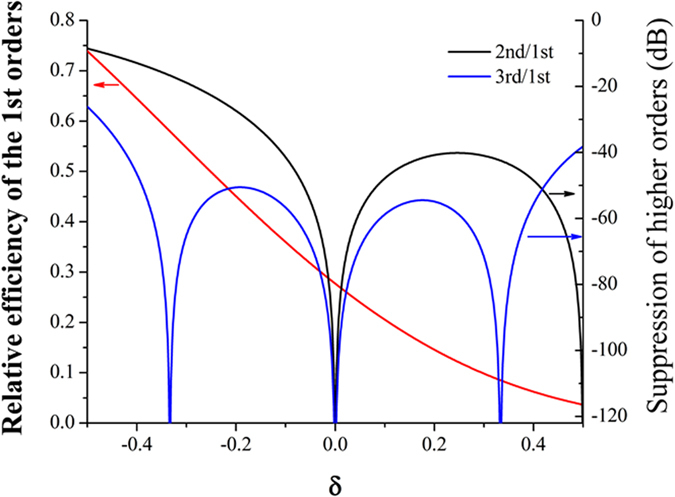
Calculated dependence of the relative efficiency of the ±1st orders and the higher-order suppression ratio on relative CD bias.

The calculated results are shown in Fig. 6. One can observe that the dispersion performance of the IRAG is strongly dependent on the CD bias of the inclined rectangular apertures. The larger feature size of the inclined rectangular aperture, the more x-ray photons it allows through, and the higher relative diffraction efficiencies of the ±1st orders are. One can also see that the relative CD bias has a much larger impact on the ±2nd orders than ±3rd orders. This is similar to the behavior of the black-white transmission gratings. To make the higher-order suppression ratio less than −46 dB (5%), the CD bias of the fabricated rectangular apertures milled into Au films needs to be controlled within ±9% of the target CD, which is slightly higher than the gate CD control budget (±10%) of both dynamic random-access memory (DRAM) and flash memory given by the international technology roadmap for semiconductors (ITRS) 2013 edition.

In conclusion, a novel scheme to realize quasi suppression of higher-order diffractions has been proposed, which is based on periodically arranged inclined rectangular apertures. Theoretical analysis reveals that the suppression ratios of the ±5th and ± 7th diffracted light are as low as −55.9 dB and −68 dB, respectively, while other higher-order diffractions approach zero. This is important for both fundamental studies and practical application of photonics. An IRAG on a quartz substrate for the visible light region and a free-standing x-ray IRAG were fabricated and characterizated, respectively. The experimental results in both the visible light and soft x-ray regions have demonstrated that this compact method can suppress the higher-order diffraction significantly. Compared with ideal sinusoidal transmission gratings, the IRAG can be fabricated by silicon planar technology. Furthermore, no support strut is needed to maintain the free-standing patterns, which is particularly suited for the extreme ultraviolet and x-ray regions. This compact method enables higher-order suppression be easily extended to other wavelength ranges of radiations, for example, microwave, infrared rays, Gamma rays, etc. The CD bias of the fabricated rectangular apertures is the dominant factor affecting the IRAG dispersion performance. To achieve higher-order suppression ratio below −46 dB, one has to control the CD bias within ±9% of target CD. The past two decades have witnessed extraordinary advances in the fabrication process of ultra large scale integrated circuits. Such fabrication process can be extended to the single optical element with higher line density and CD accuracy. Thus, this work may open up a window to new paradigms in laboratory-generated and astrophysical plasma diagnosis, synchrotron radiation x-ray monochromator, etc.

## Methods

### Fabrication process of the binary IRAG for the visible light region

Electron beam lithography and inductively coupled plasma (ICP) etching techniques were used to fabricate the binary IRAG for the visible light region. First, a chrome layer with thickness of 100 nm was deposited onto the quartz substrate with an electron beam evaporation system, and a single layer commercially electron beam resist with thickness of 600 nm was spin coated onto the chrome layer. Secondly, GDSII data of the binary IRAG were imported into an electron beam mask writer (MEBES4700S) donated by Applied Materials Company. Electron beam exposure and resist development were performed to pattern the binary IRAG onto the resist. Finally, ICP etching technique was used to transfer the resist pattern onto the chrome layer, and the residual resist was removed using plasma ashing followed by acetone rinse.

### Fabrication process of the free-standing x-ray IRAG

The free-standing x-ray IRAG fabrication process includes two key steps, i.e., fabrication of an x-ray mask and x-ray lithography. The first step is to fabricate an x-ray mask by using electron beam writing. SiC membrane with thickness of 2μm was used as substrate. A Cr (5 nm)/Au (10 nm) plating base was deposited on the substrate by using electron beam evaporation. After electron beam resist (ZEP520A) was spin coated and baked, the IRAG patterns were written in the electron beam resist using electron beam lithography system. Then the resist patterns were transferred into Au layer with thickness of 300 nm by electroplating process. The second step is to transfer the mask patterns into a positive ZEP520A resist layer upon a 0.2-μm-thick SiC membrane containing a Cr (5 nm)/Au (10 nm) plating base. The x-ray lithography was carried out at beamline 3B1B1 of Beijing synchrotron radiation facility with resist thickness of 600 nm. The resist patterns were transferred to the Au layer with thickness of 550 nm by electroplating process and the resist mold was removed using acetone followed by deionized water rinse. Finally, the Cr/Au plating base and the SiC film beneath the resist mold were removed by ion beam etching and ICP etching, respectively.

## Additional Information

**How to cite this article**: Liu, Y. *et al.* Quasi suppression of higher-order diffractions with inclined rectangular apertures gratings. *Sci. Rep.* **5**, 16502; doi: 10.1038/srep16502 (2015).

## Figures and Tables

**Table 1:** Parameters of the fabricated free-standing x-ray IRAG.

Parameters	Value
Absorber type	Au
τ (absorber thickness)	550 nm
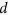 (gratings period)	1 μm
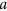 (length of the inclined rectangular aperture)	707 nm
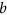 (width of the inclined rectangular aperture)	471 nm
Grating area	100 μm × 600 μm

## References

[b1] Pease L. F., Deshpande P., Wang Y., Russe W. B. & Chou S. Y.. Self-formation of sub-60-nm half-pitch gratings with large areas through fracturing. Nat. Nanotechnol. 2, 545–548 (2007). 18654365 10.1038/nnano.2007.264

[b2] Wen. H. *et al.* Subnanoradian X-ray phase-contrast imaging using a far-field interferometer of nanometric phase gratings. Nat.Commun. 4, 2659 (2013). 24189696 10.1038/ncomms3659 PMC3831282

[b3] Hurvitz. G. *et al.* Advanced experimental applications for x-ray transmission gratings spectroscopy using a novel grating fabrication method. Rev. Sci. Instrum. 83, 083109 (2012). 22938276 10.1063/1.4746771

[b4] Wang. Q. D. *et al.* Dissecting X-ray–emitting gas around the center of our galaxy. Science 30, 981–983 (2013). 23990554 10.1126/science.1240755

[b5] Miao H., Gomella A. A., Chedid N., Chen L. & Wen H.. Fabrication of 200 nm period hard X-ray phase gratings. Nano. Lett. 14, 3453–3458 (2014). 24845537 10.1021/nl5009713 PMC4055044

[b6] Eagleton R. T. & James S. F.. Transmission grating streaked spectrometer for the diagnosis of soft x-ray emission from ultrahigh intensity laser heated targets. Rev. Sci. Instrum. 75, 3969–3973 (2004).

[b7] Itou M., Harada T. & Kita T.. Soft x-ray monochromator with a varied-space plane grating for synchrotron radiation: design and evaluation. Appl. Opt. 28, 146–153 (1989). 20548441 10.1364/AO.28.000146

[b8] Born M. & Wolf. E. Principles of Optics (pergamon, London, 1980).

[b9] Filho R. L. C., Homem M. G. P, Landers R. & de Brito A. N.. Advances on the Brazilian toroidal grating monochromator (TGM) beamline. J. Electron. Spectrosc. Relat. Phenom. 144–147, 1125–1127 (2005).

[b10] He. S. *et al.* Efficiency measurement of optical components in 45–110 nm range at beamline U27, HLS. Chin. Opt. Lett. 8, 1131–1134 (2010).

[b11] Cao. L. F. *et al.* Single order x-ray diffraction with binary sinusoidal transmission grating. Appl. Phys. Lett. 90, 053501 (2007).

[b12] Jin P., Gao Y., Liu T., Li X. & Tan J.. Resist shaping for replication of micro-optical elements with continuous relief in fused silica. Opt. Lett. 35, 1169–1171 (2010). 20410955 10.1364/OL.35.001169

[b13] Takeda. Y. *et al.* X-Ray phase imaging with single phase grating. Jpn. J. Appl. Phys. 46, L89–L91 (2007).

[b14] Schattenburg M. L., Anderson E. H. & Smith H. I.. X-ray/VUV transmission gratings for astrophysical and laboratory applications. Phys. Scr. 41, 13–20 (1990).

[b15] Attwood D.. Soft X-ray and extreme Ultraviolet Radiation, Principles and Applications (Cambridge University Press, Cambridge, 1999).

[b16] Quinn F. M., Teehan D., MacDonald M., Downes S. & Bailey P.. Higher-order suppression in diffraction-grating monochromators using thin films. J Synchrotron Radiat. 5, 783–785 (1998). 15263652 10.1107/S0909049597016440

[b17] Torcal-Milla F. J., Sanchez-Brea L. M. & Bernabeu E.. Diffraction of gratings with rough edges. Opt. Express. 16, 19757–19769 (2008). 19030061 10.1364/oe.16.019757

[b18] Lee W., Lee H. & Hahn J.. Correction of spectral deformation by second-order diffraction overlapin a mid-infrared range grating spectrometer using a PbSe array detector. Infrared Phys. Technol. 67, 327–332 (2014).

[b19] Wang. C. K. *et al.* Characterization of the diffraction properties of quantum-dot-array diffraction grating. Rev. Sci. Instrum. 78, 053503 (2007). 17552816 10.1063/1.2737775

[b20] Zhang. H. P. *et al.* Elimination of higher-order diffraction using zigzag transmission grating in soft x-ray region. Appl. Phys. Lett. 100, 111904 (2012).

[b21] Kuang. L. Y. *et al.* Quasi-sinusoidal single-order diffraction transmission grating used in x-ray spectroscopy. Opt. Lett. 36, 3954–3956 (2011). 22002350 10.1364/OL.36.003954

[b22] Gao N. & Xie C.. High-order diffraction suppression using modulated groove position gratings. Opt. Lett. 36, 4251–4253 (2011). 22048381 10.1364/OL.36.004251

[b23] Meekins J. F.. Diffraction pattern of self supporting transmission gratings. Appl. Opt. 28, 1221–1227 (1989). 20548643 10.1364/AO.28.001221

[b24] Kipp. L. *et al.* Sharper images by focusing soft x-rays with photon sieves. Nature 414, 184–188 (2001). 11700552 10.1038/35102526

[b25] Miao J., Ishikawa T., Robinson I. K. & Murnane. M. M. Beyond crystallography: Diffractive imaging using coherent x-ray light sources. Science 348, 530–535 (2015). 25931551 10.1126/science.aaa1394

[b26] Heilmann. R. K. *et al.* Diffraction efficiency of 200-nm-period critical-angle transmission gratings in the soft x-ray and extreme ultraviolet wavelength bands. Appl. Opt. 50, 1364–1373 (2011). 21460902 10.1364/AO.50.001364

[b27] Xie. C. *et al.* Fabrication of x-ray diffractive optical elements for laser fusion applications. Opt. Eng. 52, 033402 (2013).

[b28] Chao W., Kim J., Rekawa S., Fischer P. & Anderson E. H.. Demonstration of 12 nm resolution Fresnel zone plate lens based soft X-ray microscopy. Opt. Express. 17, 17669–17677 (2009). 19907552 10.1364/OE.17.017669

